# Comparison of the Clinical Validity of Droplet Digital PCR to ARMS‐PCR for BRAF V600E Mutation Detection in Thyroid Nodules

**DOI:** 10.1002/jcla.23458

**Published:** 2020-07-15

**Authors:** Xiubo Li, Hong Du, Jingyan Luo, Wenshuang Ding, Bingquan Lai, Jiqian He, Shaofei Xu, Yuying Zhang

**Affiliations:** ^1^ Department of Pathology, Guangzhou First People’s Hospital School of Medicine, South China University of Technology Guangzhou China; ^2^ Forevergen Biosciences Center Guangzhou China; ^3^ Guangdong Forevergen Medical Technology Co., Ltd Foshan China; ^4^ Department of Urology The Sixth Affiliated Hospital of Guangzhou Medical University (Qingyuan People’s Hospital) Qingyuan China

**Keywords:** *BRAF* V600E, droplet digital PCR (ddPCR), fine‐needle aspiration (FNA), surgical decision‐making, thyroid nodules

## Abstract

**Objectives:**

Droplet digital PCR (ddPCR) has been reported to have a superior validity over PCR with amplification‐refractory mutation system (ARMS‐PCR) for detecting the *BRAF* V600E mutation in thyroid nodule fine‐needle aspiration (FNA) samples using cytological diagnosis as the reference. However, the added value of ddPCR on surgical decision‐making remains to be illustrated when the technique is combined with FNA cytology.

**Methods:**

A total of 277 consecutive patients with thyroid nodules were subjected to FNA cytology and *BRAF* V600E testing with ARMS‐PCR. Within this patient cohort, 90 patients underwent surgical intervention with pathological diagnosis available. *BRAF* V600E testing with ddPCR was performed retrospectively using FNA frozen DNA specimens. The clinical validity and utility of ddPCR in comparison with ARMS‐PCR were compared using surgical pathology as the reference.

**Results:**

Overall, 101 BRAF V600E mutations were detected by ddPCR, including five ARMS negative patients, four of whom were confirmed to have papillary thyroid cancer (PTC) by surgical pathology. Of the 90 patients with surgical pathology, which is considered the gold standard, ddPCR *BRAF* V600E testing yielded a sensitivity of 91.3% and specificity of 100% for PTC diagnosis, higher than that of ARMS (sensitivity 83.1%, specificity 100%). However, ddPCR only identified one more candidate patient for surgical intervention than ARMS when the techniques were combined with cytology.

**Conclusions:**

This study highlighted the superior performance of ddPCR over ARMS in *BRAF* V600E detection from thyroid nodule FNA samples. Further studies are needed to evaluate the cost‐effectiveness of replacing ARMS‐PCR with ddPCR for surgical decision‐making.

## Graphical abstract

The MicroDrop‐100^TM^ ddPCR system was able to detect the *BRAF* V600E mutation. In the representative 2‐dimention figure, the X‐axis represents the wild‐type signals (HEX) while the Y‐axis represents the mutant signals (FAM). The black points on the plot represents the negative droplets, the green points represents the droplets that are positive for wild‐type DNA only, the blue points represents the droplets that are positive for mutant DNA only, and the orange points represents the droplets that are positive for both wild‐type and mutant targets.

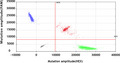

## 1 INTRODUCTION

Thyroid nodules are common, with an incident rate as high as 50%–70% in the adult population and are especially prevalent in women. The majority of thyroid nodules are benign, yet a small proportion become cancerous.^1^ Currently, ultrasound‐guided fine‐needle aspirate (FNA) cytopathology is the major method for the diagnosis of cancerous thyroid nodules.^2^ Unfortunately, the accuracy of FNA cytopathology remains unsatisfactory, with one‐third of cases categorized as being diagnostic challenging.^3^ Therefore, many patients undertook unnecessary surgery or experienced a false‐negative diagnosis due to inaccurate cytological testing.

Papillary thyroid cancer (PTC) accounts for over 80% of all thyroid cancers and has shown a dramatic increase in prevalence in recent years.^4^, ^5^ The BRAF V600E mutation occurs in 50%–89% of PTC cases and serves as an important diagnostic and prognostic biomarker.^6^, ^7^, ^8^, ^9^, ^10^ The incorporation of BRAF V600E testing has been shown to substantially improve the diagnostic accuracy of FNA.^11^, ^12^ One of the most widely used methods for BRAF V600E detection in thyroid nodules is PCR with amplification‐refractory mutation system (ARMS); this is a well‐established technique that has been widely used for rapid detection of nucleic acid mutations in a wide variety of biological samples. However, ARMS‐PCR may not be sensitive enough due to the fact that FNA samples usually have few mutant cells.^13^ Therefore, the development of a more sensitive and accurate detection method is warranted.

Droplet digital PCR (ddPCR) is a novel technology characterized by high sensitivity and absolute quantification of nucleic acid targets.^13^, ^14^ The superior sensitivity renders ddPCR as a promising detection technique in samples with trace amounts of nucleic acids, such as in liquid biopsy.^15^ The Bio‐rad QX200^TM^ ddPCR was shown to improve diagnostic accuracy for detecting the *BRAF* V600E mutation by 17% compared to cytopathology alone in thyroid nodules FNA samples.^12^ A previous study has also reported the superior sensitivity of ddPCR over ARMS‐PCR in PTC detection using the FNA cytopathology as the reference.^13^ However, as aforementioned, FNA cytological diagnosis is not accurate as surgical pathology, which is considered to be the gold standard; it remains unclear whether ddPCR has a better value than ARMS‐PCR on surgical decision‐making when in combination with FNA cytology. Therefore, in this study, we validated and compared the clinical utility of ddPCR to ARMS‐PCR for the detection of the *BRAF* V600E mutation in FNA specimens of thyroid nodules, using pathological diagnosis following surgery as the gold standard.

## 2 MATERIALS AND METHODS

### 2.1 Study subjects and FNA samples collection

This is a retrospective cohort study involved consecutive patients who underwent FNA cytology and *BRAF* V600E testing with ARMS for thyroid nodules in The Guangzhou First People's Hospital between October 2018 and July 2019. Based on the thyroid ultrasound findings, high‐risk individuals were referred to ultrasound‐guided FNA biopsy performed by trained physicians according to the recommended guideline.^16^ In addition to cytopathological examination, FNA specimens were also collected for BRAF V600E mutation testing. The pathologists who made the cytopathological diagnosis were blind to the ARMS‐PCR and ddPCR results. The study was approved by the institutional ethical review board at The Guangzhou First People's Hospital, and patients gave informed consent.

### 2.2 Bethesda classification and surgical intervention

The Bethesda classification system is used for reporting FNA cytology. Based on this scheme, cases were divided into six categories: (I) nondiagnostic or unsatisfactory; (II) benign; (III) atypia of undetermined significance or follicular lesion of undetermined significance; (IV) follicular neoplasm or suspicious for a follicular neoplasm; (V) suspicious for malignancy; and (VI) malignant.^17^

ARMS‐PCR detection of *BRAF* V600E is the routine procedure for thyroid nodule FNA samples in the hospital. Patients with suspected cancer were referred to surgical intervention based on positive findings on ARMS *BRAF* V600E testing, FNA Bethesda category V/VI, and clinical consideration by the physicians. In total, surgery was performed in 90 cases and pathological diagnosis was used as the gold standard. The ddPCR test was performed retrospectively in September 2019 using the frozen FNA DNA specimens; therefore, the ddPCR findings were not used for making treatment decisions in this study.

### 2.3 Nucleic acid extraction from FNA samples

The Tissue DNA Extration Kit (AmoyDx, China) was used for DNA extraction from thyroid nodule FNA samples. After collection, FNA specimens were immediately transferred to a 1.5 mL microcentrifuge tube with 180 μL lysis buffer and DNA was extracted according to the manufacture's protocol. OD 260/280 was used to measure the DNA concentration.

### 2.4 Plasmid preparation

The wild‐type *BRAF* plasmid and the V600E mutant plasmid were first synthesized and then had their sequences confirmed by Generay Biotech Co., Ltd (Shanghai). Mutant and wild‐type plasmids were prepared as the positive and negative controls. The QIAamp DNA mini kit (QIAGEN) was used for DNA extraction from plasmids. Qubit 4.0 (Thermo Fisher) was used to determine the concentration of plasmid DNA. Mutant plasmid DNA was diluted with wild‐type plasmid DNA in a series of concentrations (0.05%, 0.1%, 0.5%, 1%, 5%, 10%, and 50%) to determine the sensitivity of ddPCR for *BRAF* V600E detection.

### 2.5 *BRAF* V600E mutation detection with ARMS‐PCR and ddPCR

The ARMS‐PCR assay was performed on an ABI 7500 Real‐time PCR system (Life Technologist) with a *BRAF* V600E Diagnostic Kit (AmoyDx) according to the manufacturer's protocol. The details of this testing system have been published elsewhere.^13^

The MicroDrop‐100^TM^ ddPCR system (Forevergen), which is based on water‐emulsion droplet technology, was used for the ddPCR assay. The primers and probes used for *BRAF* V600E detection with ddPCR are as follows:

5′‐TGTTTTCCTTTACTTACTACACCTCAGA‐3′ (forward);

5′‐TGACAACTGTTCAAACTGATGGGAC‐3′ (reverse);

FAM‐CTAGCTACAGAGAAATC‐MGB (mutant probe);

VIC‐CTAGCTACAGTGAAATC‐MGB (wild‐type probe).

The ddPCR assays were carried out in 20 μL reaction mixtures containing 10 μL ddPCR Supermix for Probes (Forvergen), 2 μL 10 × *BRAF* V600E Mutant Assay (Forvergen), DNA templates, and deionized water. Droplets were generated using the MicroDrop‐100A^TM^ Droplet Generator (Forvergen) and then were moved into 96‐well cartridges according to manufacturer's instructions. Amplifications were performed using the following conditions: 1 cycle of 95°C for 10 minutes, 45 cycles alternating between 95°C for 30 s and 60°C for 1 min, and 1 cycle of 98°C for 10 min before holding at 16°C. After amplification, the 96‐well cartridges were placed into a MicroDrop‐100B^TM^ detector (Forevergen) to measure the fluorescence signals. The mutation abundance for each sample was calculated with QuantDrop analysis software (Forevergen) following the principle of the Poisson distribution. The sample was regarded as a positive *BRAF* V600E mutation if there were three or more positive droplets.

The ARMS‐PCR assay and the ddPCR assay were performed separately by two researchers to avoid mutual influences.

### 2.6 Statistical analysis

Analysis was performed using the SPSS 20.0 software (IBM, USA). We used chi‐square tests for comparisons between groups for categorical variables and two‐tailed t tests for continuous variables. A *P* value < 0.05 was considered statistically significant.

## 3 RESULTS

### 3.1 Cytological findings of FNA specimens

A total of 277 patients with both FNA cytology and ARMS‐PCR *BRAF* V600E testing available were enrolled in this study. The mean age (±standard deviation) was 44.9 ± 13.2 years (range: 13‐80), and 79.1% of the cohort (n = 219) was female. The cytopathology of FNA indicated that 86 cases (31.0%) were classified as malignant tumor (category VI) and 18 (6.5%) cases were suspicious for malignancy (category V), while 18 (6.5%) cases were classified as category III/IV with diagnostic challenge, and 43 (15.5%) cases were nondiagnostic (category I). The detailed description of the Bethesda classification is shown in Table 1.

**Table 1:** Clinical and molecular characteristics of participants

		High‐risk cases^a^ (n = 125)		
	Total (n = 277)	with surgery (n = 90)	Loss to surgical follow‐up (n = 35)	*P* value
Age, years	44.9 ± 13.2	43.1 ± 12.5	41.8 ± 13.1	.597
Sex				.688
Male	58(20.9)	21 (23.3)	7 (20.0)	
Female	219 (79.1)	69 (76.7)	28 (80.0)	
TI‐RADS grading				.648
NA	9 (3.2)	‐	‐	
1 ~ 2	4 (1.4)	1 (1.1)	0	
3	48 (17.3)	5 (5.6)	2 (5.9)	
4	199 (71.8)	70 (77.8)	29 (85.3)	
5	12 (4.3)	9 (10.0)	3 (8.8)	
6	5 (1.8)	5 (5.6)	0	
Nodules number				
NA	4 (1.4)			.27
1	86 (31.0)	37 (41.1)	9 (26.5)	
2	36 (13.0)	16 (17.8)	6 (17.6)	
≥3	151 (54.5)	37 (41.1)	19 (55.9)	
Bethesda classification for FNA cytology				.974
I: ND/UNS	43 (15.5)	6 (6.7)	2 (5.7)	
II: benign	112 (40.4)	5 (5.6)	2 (5.7)	
III: AUS/FLUS	16 (5.8)	4 (4.4)	1 (2.9)	
IV: FN/SFN	2 (0.7)	1 (1.1)	0	
V: SM	18 (6.5)	12 (13.3)	6 (17.1)	
VI: malignancy	86 (31.0)	62 (68.9)	24 (68.6)	
*BRAF* V600E mutation				
ARMS‐PCR positive	96 (34.7)	69 (76.7)	27 (77.1)	1.00
ddPCR positive	101 (36.5)	73 (81.1)	28 (80.0)	.887

Note

TI‐RADS: Thyroid Imaging Reporting and Data System (TI‐RADS), 1: normal thyroid gland; 2: benign conditions (0% risk of malignancy); 3: probably benign nodules (<5% malignancy); 4: suspicious nodules (5%‐80% malignancy); 5: probably malignant nodules (>80% malignancy); 6: biopsy‐proven malignancy.

Bethesda classification: I, specimens nondiagnostic/unsatisfactory (ND/UNS); II, benign; III, atypia of undetermined significance/follicular lesion of undetermined significance (AUS/FLUS); IV, follicular neoplasm/suspicious for a follicular neoplasia (FN/SFN); V, suspicious for malignancy (SM); VI, malignancy.

Abbreviations: FNA, fine‐needle aspirate; NA, not available.

^a^ High‐risk cases were defined as a patient with Bethesda category V/VI on cytopathology, or positive BRAF V600E mutation detected by ARMS, or those referred to surgery by the clinical judgment of physicians.

### 3.2 The sensitivity of *BRAF* V600E mutation testing with ddPCR

**Figure 1:**
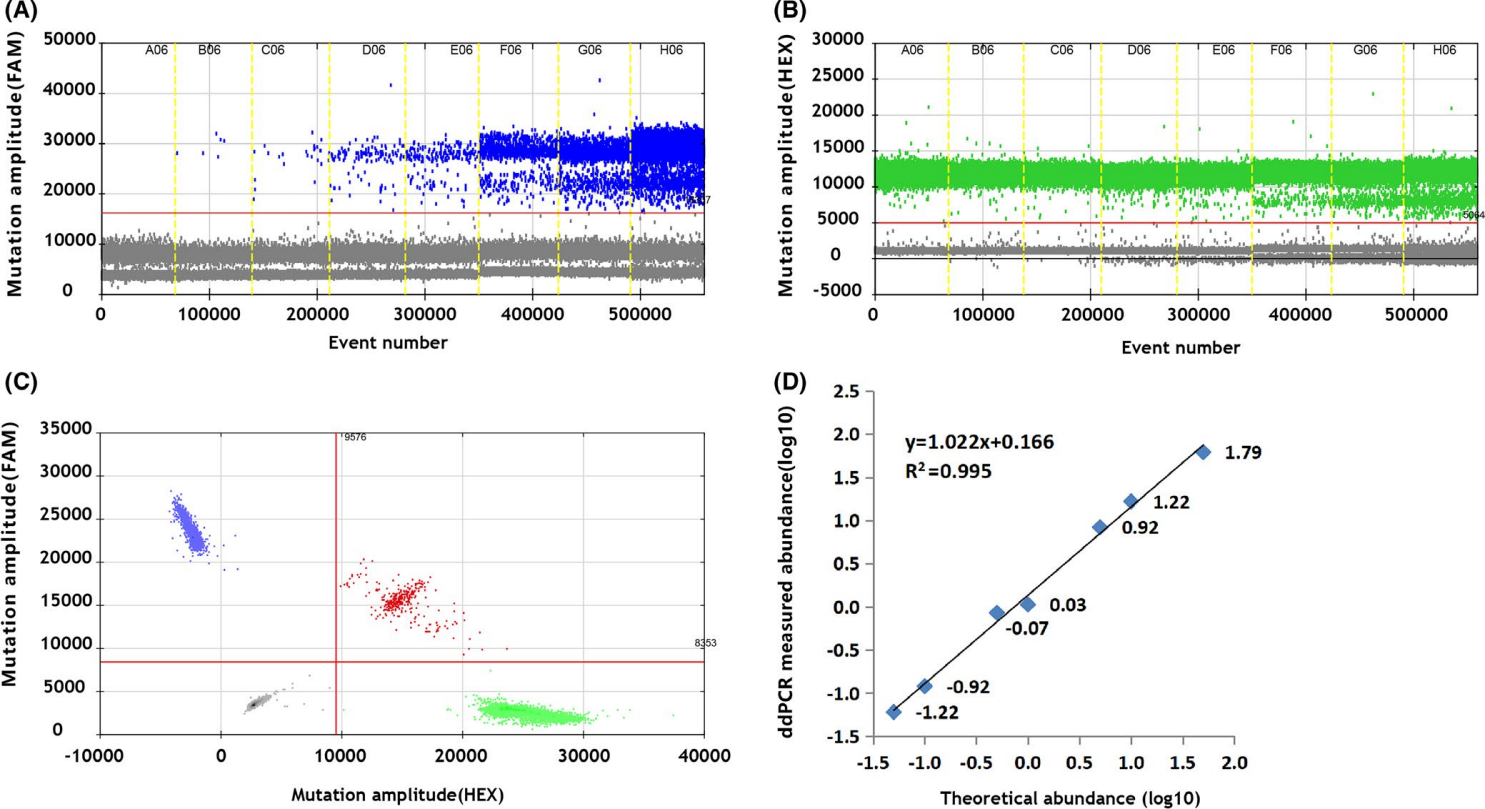
Representative 1D, 2D, and fractional abundance figures of ddPCR. ddPCR was used to detect the *BRAF* V600E mutation. FAM, mutant probe; HEX, wild‐type probe. A. & B,. Representative 1D figure showing FAM and HEX fluorescent signals. C,. In the representative 2D figure, the X‐axis represented the wild‐type signals (HEX) while the Y‐axis represented the mutant signals (FAM). D,. Fractional abundances of plasmid dilutions were calculated with QuantaSoft analysis software, and linearity of ddPCR detecting the *BRAF* V600E mutation was calculated (*R* ^2^ = .995, *y* = 1.022*x* + 0.166). The X‐axis represents the theoretical abundance of plasmid dilutions of 0.05%, 0.1%, 0.5%, 1.0%, 5.0%, and 10% (log_10_ transformed value), while the Y‐axis represents the ddPCR‐measured abundance (log_10_ transformed value)

To evaluate the sensitivity of ddPCR, the *BRAF* V600E mutant plasmid was diluted with wild‐type plasmids to V600E concentrations of 0.05%, 0.1%, 0.5%, 1%, 5%, 10%, and 50%. As shown in Figure 1, ddPCR was able to detect the *BRAF* V600E mutation at all dilutions and the calculated lowest copy number of the sample was approximately 1‐2 copies/20 μl (0.05%). Additionally, the ddPCR results were highly reproducible in various *BRAF* V600E mutation dilutions: CV_0.1% _= 9.38%, CV_1% _= 5.81%, and CV_10%_ = 0.99%.

### 3.3 ddPCR vs. ARMS‐PCR for *BRAF* V600E mutation detection

Using ARMS, we detected 96 (34.7%) cases with the *BRAF* V600E mutation, including 14 cases classified as Bethesda I‐III, and 82 cases classified as Bethesda V‐VI. Compared to ARMS, ddPCR detected five more mutant cases, of whom four cases were subjected to surgical intervention and PTC was confirmed by surgical pathological diagnosis, suggesting ddPCR has a higher sensitivity than ARMS on FNA *BRAF* V600E detection (Tables 2, 3). Additionally, ddPCR detected the absolute quantity of *BRAF* V600E copies, which ranged from 6 to 5720 (0.05% to 43.4%) in the 20ul reaction system.

**Table 2:** Comparison of ARMS‐PCR and ddPCR in *BRAF* V600E detection in thyroid nodule fine‐needle aspirate (n = 277)

ARMS‐PCR	ddPCR			*P* value
	Negative	Positive	Total	
Negative	176	**5**	181	<.0001
Positive	0	96	96	
Total	176	101	277	

**Table 3:** The clinical validity of five patients with inconsistent ARMS‐PCR and ddPCR findings

Patient ID	Mutation rate (positive copies/20μl)	ddPCR	ARMS‐PCR	Ultrasonic TI‐RADS	Cytology Bethesda grading	Surgical pathology
23	0.05% (6)	+	‐	4c	III	Papillocarcinoma
48	0.18% (6)	+	‐	4b	V	NA
104	0.02% (12.6)	+	‐	5	VI	Papillocarcinoma
112	0.06% (10.2)	+	‐	4b	VI	Papillocarcinoma
117	24.79% (134)	+	‐	4b	VI	Papillocarcinoma

Abbreviations: NA, not available; TI‐RADS, Thyroid imaging reporting and data system.

### 3.4 FNA cytopathology, ARMS, and ddPCR *vs*. surgical pathology in a subgroup of patients

Of the 277 participants, 90 high‐risk cases who had a positive finding on ARMS‐PCR *BRAF* V600E and/or were classified as Bethesda category V/VI undertook surgical intervention. However, 35 high‐risk cases with positive ARMS findings and/or Bethesda categories V/VI were lost to follow‐up for surgical intervention. Nonetheless, the demographic and clinical characteristics were similar in the high‐risk patients with or without surgical intervention (Table 1).

We compared the clinical validity of ARMS, ddPCR, and FNA cytology in this subgroup of patients using surgical pathology as the gold standard. As shown in Table 4, 83 out of 90 patients were diagnosed with PTC by pathological examination, including four cases diagnosed as benign (Bethesda II) by FNA cytopathology. In the subgroup of patients categorized as Bethesda V/VI, FNA cytology showed 86.7% sensitivity and 71.4% specificity, with positive predictive value (PPV) of 97.3% and negative predictive value (NPV) of 25%. In contrast, all cases with the *BRAF* V600E mutation detected by ARMS and/or ddPCR were confirmed to have PTCs by pathological diagnosis, yielding a 100% specificity and 100% PPV for PTC diagnosis. Compared to ARMS, ddPCR alone had higher sensitivity and NPV. Additionally, when ddPCR was used to test patients categorized as Bethesda V/VI, the sensitivity reached up to 98.8% with 71.4% specificity, and only three cases were incorrectly diagnosed, confirming the superior clinical validity of ddPCR in *BRAF* V600E testing over ARMS.

### 3.5 The ddPCR *BRAF* V600E mutation rate by age, sonographic grade, and FNA cytology

**Figure 2:**
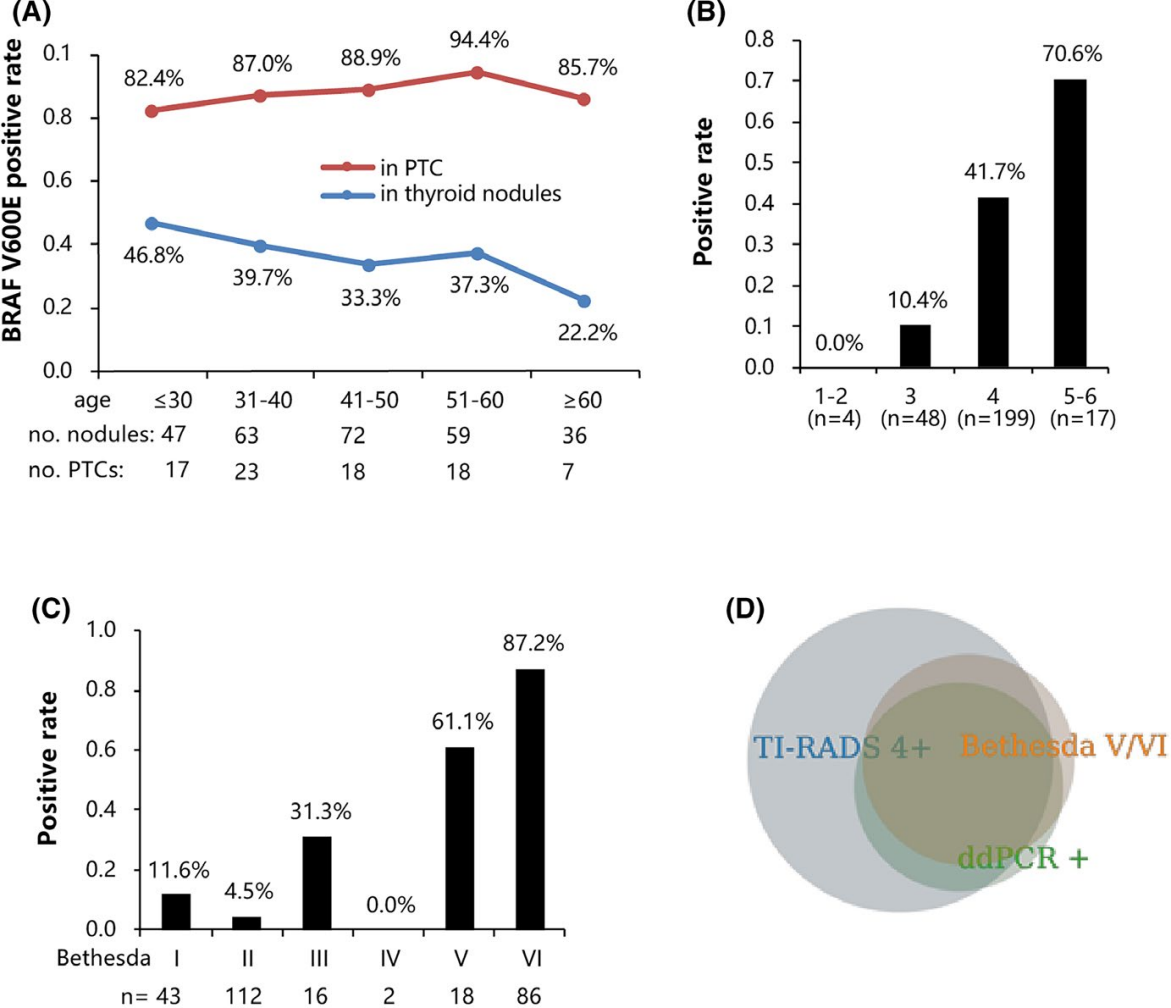
*BRAF* V600E mutation rate measured by ddPCR. A, The ddPCR‐measured *BRAF* V600E mutation rate by different age groups. The blue line indicates the *BRAF* V600E mutation rate in thyroid nodules, while the red line indicates the *BRAF* V600E mutation rate in those with surgical pathology‐confirmed thyroid cancer (both *P* for trend < 0.05). B &C, The ddPCR‐measured *BRAF* V600E mutation rate by ultrasonic TI‐RADS grades and Bethesda categories of cytopathology. D,. The Venn diagram showing the relationship of the *BRAF* V600E mutation rate screened by thyroid ultrasound image TI‐RADS grade 3 or above, Bethesda (V/VI) and ddPCR

To evaluate the association of the *BRAF* V600E mutation with age, we determined the V600E mutation rate by age group. Although older PTC patients were more likely to have the *BRAF* V600E mutation, *BRAF* V600E was more prevalent in younger individuals with thyroid nodules detected with ddPCR; both correlate significantly with aging (both *P* for trend < 0.05) (Figure 2A).

Unsurprisingly, *BRAF* V600E mutations were more prevalent in individuals with higher TI‐RADS and FNA Bethesda grades. Nevertheless, even in those with TI‐RADS grade 3 or lower in the ultrasonic examination, there were still five (11%) patients with the *BRAF* V600E mutation. Moreover, in patients categorized as Bethesda I, II, and III, the *BRAF* V600E mutation rates were 12%, 4%, and 31%, respectively. These patients were most likely to experience false‐negative diagnosis if only cytology was used, as was illustrated in the Venn diagram (Figure 2B‐D).

## 4 DISCUSSION

The *BRAF* V600E mutation testing on FNA specimens from thyroid nodules greatly improves diagnostic accuracy.^11^, ^12^ However, detecting trace amounts of DNA molecules in FNA specimens remains challenging. DdPCR is a cutting‐edge technique that enables the sensitive and accurate detection of molecular markers from samples with a limited amount of target DNA.^18^ In this study, we confirmed the clinical validity of the MicroDrop‐100^TM^ ddPCR system. It showed a better performance than ARMS‐PCR in *BRAF* V600E mutation detection on thyroid nodule FNA specimens, which is in line with a previous study.^13^ The combination of ddPCR and FNA cytology may serve as a better option for the diagnosis of thyroid nodules.

In this study, ddPCR detected five more mutant specimens than ARMS‐PCR, three of which displayed mutation rates of less than 0.1%, illustrating the ultra‐sensitivity of ddPCR. In support of this finding, four of these patients were confirmed to have PTC by pathological diagnosis following thyroidectomy. It is worth noting that one of these patients was classified as Bethesda category III; therefore, he or she might have been misdiagnosed if the decision was simply made according to FNA cytopathology and ARMS. Overall, our findings highlight the superior sensitivity of ddPCR over ARMS in *BRAF* V600E detection in thyroid FNA specimens, especially when only trace amounts of DNA molecules are in the sample, which is consistent with a previous report.^13^ However, it is also worth noting that ddPCR only identified one more candidate patient than ARMS when the technique was used in combination with Bethesda V/VI as the screening scheme for further surgical intervention. Thus, further studies with a larger sample size are still warranted to illustrate the cost‐effectiveness of replacing ARMS with ddPCR as a companion test for FNA cytopathology.

The Bethesda classification system is the most widely used scheme for reporting FNA cytopathology. According to this scheme, surgical interventions are usually needed for patients with category V (suspicious for malignancy) and VI (malignant), due to the high probability for malignancy in these cases.^17^ Although only 6.5% of the cases in our study were classified as category III/IV, such cases are diagnostically challenging and diagnostic surgery may be required according the 2017 Bethesda guideline ^17^; however, less than one‐third of these cases were tested *BRAF* V600E positive in our study. Since *BRAF* V600E had 100% specificity for PTC according to the subgroup analysis using surgical pathology as the gold standard, two‐thirds of these patients with category III/IV might have benign lesions and hence surgeries might not be necessary. Our findings are consistent with previous studies which also reported that less than one‐third of patients with category III/IV were harboring cancer.^19^, ^20^, ^21^ Additionally, in this study, over half of thyroid nodules were classified in category I (undiagnosed) and II (benign) and the *BRAF* V600E mutation rates were 12% and 4%, respectively, with similar malignancy rates reported in previous studies.^21^ These malignant cases with category I/II would probably be missed if diagnosis was simply based on FNA cytology. Given the huge number of patients with thyroid nodules, even a small difference in the misdiagnosis rate might translate into a tremendous burden on the healthcare system. Therefore, our data warrant the combination of *BRAF* V600E mutation testing to improve the diagnostic accuracy for the early detection of thyroid cancer.

The current study found a higher *BRAF* V600E mutation rate in younger people, with a significant decreasing trend over aging, in line with the fact that PTCs are most likely to occur in young women.^5^ In the UK, it has been reported that the incidence rates of thyroid cancer in women reach a peak at ages 35‐39 years and then decline steadily.^5^ In the United States, the analysis of the Surveillance, Epidemiology, and End Results‐9 (SEER‐9) cancer registry program revealed the highest thyroid cancer incidence rate at ages 40‐59 years.^4^ Nevertheless, the current study identified the highest *BRAF* V600E mutation rate in ages around 30 years, suggesting an earlier onset age of thyroid cancer in Chinese women. Considering that young people constitute the majority of the workforce, the higher *BRAF* V600E mutation rate calls for an immediate initiative to develop more sensitive testing to improve early diagnosis. Additionally, over 80% of patients with PTCs were harboring the *BRAF* V600E mutation in this study, with a higher mutation rate in older patients, consistent with previous investigations y.^9^, ^22^ It remains unclear why older PTC patients tend to have a higher *BRAF* V600E mutation rate, although old people have a significantly lower risk for PTC than young people. We speculate that PTCs in young people might be more genetically heterogeneous and genetic risk predisposition other than the *BRAF* V600E mutation contributes to the earlier onset of thyroid carcinoma in these patients.

There are several major limitations in this study. Firstly, in our study, only high‐risk individuals were referred to FNA biopsy and surgery; therefore, the diagnostic validity parameters, including sensitivity and specificity, were probably biased to a better level. Secondly, we did not perform post‐operative testing to confirm the pre‐operative *BRAF* V600E testing. Thirdly, it is worth noting that samples with low amounts of mutant copies still have a chance of a false‐negative error. Lastly, this is a single center study with a limited sample size; therefore, the clinical utility of ddPCR in *BRAF* V600E testing should be confirmed in a larger population.

## 5 CONCLUSION

In spite of the limitations, this is the first study that compared the clinical validity of ddPCR to ARMS‐PCR in *BRAF* V600E detection on FNA specimens from thyroid nodules using the pathological diagnosis following surgery as the gold standard. The ddPCR technique demonstrated superior performance over ARMS‐PCR, particularly in trace amounts of target DNA specimens. However, it is also worth noting that compared to ARMS plus cytology, ddPCR plus cytology only identified one more candidate patient for surgical intervention. Therefore, further investigations with larger sample sizes and cost‐effectiveness evaluation are needed to find out whether such slight superiority can be translated into substantial benefit to the healthcare system due to the extremely high prevalence of thyroid nodules.

## CONFLICT OF INTEREST

The authors declare no conflict of interest.

## AUTHOR CONTRIBUTIONS

X.L, S.X, and Y.Z conceived the study and its design. X.L, H.D, and W.D were involved in the sample collection, reviewed, and edited the study. X.L, J.L, and J.H contributed to the testing, data analysis, and reviewed the study. X.L and Y.Z wrote the study. S.Z and YZ contributed to the discussion, and reviewed, edited, and finalized the study. All the authors read and approved the final study.

6

**Table 4:** Diagnostic performance of cytology, ARMS‐PCR and ddPCR in patients with pathological diagnosis following surgery (n = 90)

	Surgical pathology					
	Negative (n = 7)	Positive (n = 83)	Sensitivity	Specificity	PPV	NPV
Bethesda classification						
I	3	3				
II	1	4				
III‐IV	1	4				
V‐VI	2	72	86.7%	71.4%	97.3%	25.0%
ARMS‐PCR						
Negative	7	14				
Positive	0	69	83.1%	100%	100%	33.3%
ddPCR						
Negative	7	10				
Positive	0	73	91.3%	100%	100%	41.2%
Bethesda V/VI + ARMS‐PCR						
Negative	5	2				
Positive	2	**81**	97.6%	71.4%	97.6%	71.4%
Bethesda V/VI + ddPCR						
Negative	5	1				
Positive	2	**82**	98.8%	71.4%	97.6%	83.3%

Note

Bethesda classification: I, specimens nondiagnostic/unsatisfactory (ND/UNS); II, benign; III, atypia of undetermined significance/follicular lesion of undetermined significance (AUS/FLUS); IV, follicular neoplasm/suspicious for a follicular neoplasia (FN/SFN); V, suspicious for malignancy (SM); VI, malignancy.

Abbreviations: NPV, negative predictive value; PPV, positive predictive value.

## References

[1] Dean DS , Gharib H . Epidemiology of thyroid nodules. Best Pract Res Clin Endocrinol Metab. 2008;22:901‐911. 19041821 10.1016/j.beem.2008.09.019

[2] Haugen BR , Alexander EK , Bible KC , et al. 2015 American Thyroid Association Management Guidelines for Adult Patients with Thyroid Nodules and Differentiated Thyroid Cancer: The American Thyroid Association Guidelines Task Force on Thyroid Nodules and Differentiated Thyroid Cancer. Thyroid. 2016;26:1‐133. 26462967 10.1089/thy.2015.0020 PMC4739132

[3] Paschke R , Cantara S , Crescenzi A , Jarzab B , Musholt TJ , Sobrinho SM . European Thyroid Association Guidelines regarding Thyroid Nodule Molecular Fine‐Needle Aspiration Cytology Diagnostics. Eur Thyroid J. 2017;6:115‐129. 28785538 10.1159/000468519 PMC5527175

[4] Lim H , Devesa SS , Sosa JA , Check D , Kitahara CM . Trends in Thyroid Cancer Incidence and Mortality in the United States, 1974–2013. JAMA. 2017;317:1338‐1348. 28362912 10.1001/jama.2017.2719 PMC8216772

[5] UK National Cancer Intelligence Network . Thyroid cancer – trends by sex, age and histological type: NCIN Data Briefing. http://www.ncin.org.uk/publications/data_briefings/thyroid_cancer_trends_by_sex_age_and_histological_type (accessed on May 31 2020).

[6] Liu L , Chang JW , Jung SN , et al. Clinical implications of the extent of BRAF(V600E) alleles in patients with papillary thyroid carcinoma. Oral Oncol. 2016;62:72‐77. 27865374 10.1016/j.oraloncology.2016.10.005

[7] Jinih M , Foley N , Osho O , et al. BRAF(V600E) mutation as a predictor of thyroid malignancy in indeterminate nodules: A systematic review and meta‐analysis. Eur J Surg Oncol. 2017;43:1219‐1227. 27923591 10.1016/j.ejso.2016.11.003

[8] Lee JH , Lee ES , Kim YS . Clinicopathologic significance of BRAF V600E mutation in papillary carcinomas of the thyroid: a meta‐analysis. Cancer. 2007;110:38‐46. 17520704 10.1002/cncr.22754

[9] Kebebew E , Weng J , Bauer J , et al. The prevalence and prognostic value of BRAF mutation in thyroid cancer. Ann Surg. 2007;246:466‐470. 17717450 10.1097/SLA.0b013e318148563d PMC1959359

[10] Zhang YL , Wang DQ , Zhang H , et al. The value of BRAF V600E gene detection in thyroid cytological diagnosis via a large population. Zhonghua Bing Li Xue Za Zhi. 2020;49:186‐188. 32074736 10.3760/cma.j.issn.0529-5807.2020.02.017

[11] Moses W , Weng J , Sansano I , et al. Molecular testing for somatic mutations improves the accuracy of thyroid fine‐needle aspiration biopsy. World J Surg. 2010;34:2589‐2594. 20703476 10.1007/s00268-010-0720-0 PMC2949559

[12] Biron VL , Matkin A , Kostiuk M , et al. Analytic and clinical validity of thyroid nodule mutational profiling using droplet digital polymerase chain reaction. J Otolaryngol Head Neck Surg. 2018;47:60. 30249281 10.1186/s40463-018-0299-2 PMC6154415

[13] Xu X , Ma X , Zhang X , et al. Detection of BRAF V600E mutation in fine‐needle aspiration fluid of papillary thyroid carcinoma by droplet digital PCR. Clin Chim Acta. 2019;491:91‐96. 30682328 10.1016/j.cca.2019.01.017

[14] Pinheiro LB , Coleman VA , Hindson CM , et al. Evaluation of a droplet digital polymerase chain reaction format for DNA copy number quantification. Anal Chem. 2012;84:1003‐1011. 22122760 10.1021/ac202578x PMC3260738

[15] Hiemcke‐Jiwa LS , Minnema MC , Radersma‐van Loon JH , et al. The use of droplet digital PCR in liquid biopsies: A highly sensitive technique for MYD88 p.(L265P) detection in cerebrospinal fluid. Hematol Oncol. 2018;36(2):429‐435. 29210102 10.1002/hon.2489

[16] Thyroid Surgeon Committee of the Chinese Physicians Association Surgeons Branch . Ultrasound‐guided fine needle aspiration biopsy expert consensus and operation guideline (version 2018). Chinese J Prac Surg. 2018;38:241‐244.

[17] Cibas ES , Ali SZ . The 2017 Bethesda System for Reporting Thyroid Cytopathology. Thyroid. 2017;27:1341‐1346. 29091573 10.1089/thy.2017.0500

[18] Taylor SC , Laperriere G , Germain H . Droplet Digital PCR versus qPCR for gene expression analysis with low abundant targets: from variable nonsense to publication quality data. Sci Rep. 2017;7:2409. 28546538 10.1038/s41598-017-02217-x PMC5445070

[19] Ho AS , Sarti EE , Jain KS , et al. Malignancy rate in thyroid nodules classified as Bethesda category III (AUS/FLUS). Thyroid. 2014;24:832‐839. 24341462 10.1089/thy.2013.0317 PMC4948206

[20] Godoi Cavalheiro B , Kober Nogueira Leite A , Luongo de Matos L , et al. Malignancy rates in thyroid nodules classified as bethesda categories III and IV: Retrospective data from a tertiary. Center. Int J Endocrinol Metab. 2018;16:e12871. 10.5812/ijem.12871 PMC5903393 29696036

[21] Thewjitcharoen Y , Butadej S , Nakasatien S , et al. Incidence and malignancy rates classified by The Bethesda System for Reporting Thyroid Cytopathology (TBSRTC) ‐ An 8‐year tertiary center experience in Thailand. J Clin Transl Endocrinol. 2019;16:100175. 30815363 10.1016/j.jcte.2018.12.004 PMC6378904

[22] Trovisco V , Soares P , Preto A , et al. Type and prevalence of BRAF mutations are closely associated with papillary thyroid carcinoma histotype and patients' age but not with tumour aggressiveness. Virchows Arch. 2005;446:589‐595. 15902486 10.1007/s00428-005-1236-0

